# Epilepsy in Wolf–Hirschhorn Syndrome: Clinical Insights from a Pediatric Cohort and a Review of the Literature

**DOI:** 10.3390/jcm14228044

**Published:** 2025-11-13

**Authors:** Raquel Blanco-Lago, Ignacio Málaga, Jair Antonio Tenorio-Castaño, Nelly Álvarez-Álvarez, Pablo Lapunzina, Julián Nevado

**Affiliations:** 1 Neuropediatrics Service, Universitary Hospital Central de Asturias (HUCA), 33011 Oviedo, Spain; neuropediatria.huca@gmail.com (I.M.); nellyavz@gmail.com (N.Á.-Á.); 2 Medical and Molecular Genetics Institute (INGEMM)-IdiPAZ, Universitary Hospital of La Paz, 28046 Madrid, Spain; jair.tenorio@salud.madrid.org (J.A.T.-C.); plapunzina@gmail.com (P.L.); 3 CIBERER—Biomedical Research Network Centre in Rare Diseases, National Health Institute Carlos III (ISCIII), 28046 Madrid, Spain; 4 ITHACA-European Reference Network-Hospital la Paz, 28046 Madrid, Spain; 5 Facultad HM de Ciencias de la Salud, Universidad Camilo José Cela, 28015 Madrid, Spain

**Keywords:** Wolf–Hirschhorn syndrome, epilepsy, antiseizure medications, pediatric cohort, 4p16.3

## Abstract

**Background:** Wolf–Hirschhorn syndrome (WHS) is a rare contiguous gene deletion disorder associated with a high incidence of early-onset epilepsy. Despite the clinical relevance of seizures in this population, few large-scale studies have provided detailed data on epilepsy phenotypes and treatment outcomes. **Methods:** We analyzed a cohort of 140 individuals with WHS from Spain and Latin America. Using validated caregiver-reported questionnaires, we collected detailed information on seizure types, antiseizure medications (ASM) use, and associated comorbidities. Statistical comparisons were made to identify correlations between epilepsy severity, deletion size, and functional outcomes. **Results:** Epilepsy was observed in 92% of patients, typically beginning before 12 months of age. Multiple seizure types were common, particularly generalized tonic–clonic and atypical absence seizures. Status epilepticus occurred in 58% of cases, with a high proportion requiring multiple ASMs. Valproic acid and levetiracetam were the most commonly used treatments. Patients with more severe epilepsy tended to have larger deletions (>9 Mb) and poorer developmental outcomes. ASM discontinuation was significantly associated with older age at evaluation, supporting improved seizure control over time. **Conclusions:** Epilepsy in WHS is frequent, often severe in early childhood, and associated with neurodevelopmental impairment and increased treatment burden. While some patients show improvement with age, early aggressive management using appropriate ASMs may be critical to improve neurological prognosis. Advances in diagnosis, early intervention, and targeted therapies offer hope for improved long-term outcomes in this vulnerable population.

## 1. Introduction

Wolf–Hirschhorn syndrome (WHS; OMIM #194190) is a contiguous gene syndrome resulting from deletions involving the short arm of chromosome-4 (region 4p16.3) [1,2,3]. Affected individuals typically share a recognizable phenotype (see Table 1, adapted from the cohort analyzed herein), including the characteristic “Greek warrior helmet” facial appearance, intrauterine and postnatal growth restriction, global developmental delay, and seizures. Additional clinical features may also be variably presented [1,2,3,4,5,6]. Among these, epilepsy is one of the most impactful conditions affecting quality of life in WHS, especially during early infancy (6–12 months) [7,8,9]. Seizures may be life threatening in some cases due to the high incidence of status epilepticus. In addition, inadequate seizure control has been strongly associated with poorer neurodevelopmental outcomes [2,3,4,5,6,7].

The estimated incidence of WHS is 1 in 50,000 live births, with a reported female predominance (2:1). However, this figure likely underestimates the true prevalence, particularly in milder phenotypes that go undiagnosed [1,2,3,4]. In fact, a study from our group has revealed a considerably lower prevalence of WHS in Spain, supporting the hypothesis of misdiagnosis [11].

Putative genotype–phenotype correlations have been proposed in WHS, whereby individuals with larger deletions tend to exhibit more severe clinical features, including also more severe epilepsy [4,5,9,10]. In addition, epilepsy in WHS often coexists with other comorbidities, such as developmental delay, hypotonia, nephropathy, congenital heart disease, failure to thrive, swallowing difficulties, and behavioral disturbances. These comorbidities complicate epilepsy management and necessitate a holistic, personalized approach to treatment selection, considering the seizure type and the broader clinical context. Epilepsy affects up to 90–95% of individuals with WHS and typically begins in early infancy (mean onset at 9 months, before 2 years of age). Seizures are frequently drug-resistant, although seizure control seems to tend to improve with age [5,6,12]. Some patients show seizure sensitivity to fever and poor response to certain antiseizure medications (ASMs), such as carbamazepine (CBZ), oxcarbazepine (OXC), and phenytoin (DPH) [12,13,14]. The status epilepticus is a common feature, especially in early childhood [7,8,9,10,12,13,14]. Furthermore, a higher number of episodes of status has been suggested to be associated with a more pronounced psychomotor delay [3,4,5,6].

The underlying molecular mechanisms responsible for epilepsy in WHS seemed complex and remain poorly understood. Several candidate genes have been proposed, located both within the WHS critical region and toward the telomeric end of 4p [5,6,9,15]. In fact, current models favor a multigene mechanism involving synergistic interactions between multiple genes and molecular pathways, including epigenetic factors [5,13,14,15,16]. One compelling hypothesis implicates the *PIGG* gene, frequently deleted in WHS, in GPI (Glycosylphosphatidylinositol)-anchor biosynthesis [16]. Disruption of this pathway may help to explain the observed resistance to sodium channel-targeting ASMs in these patients. In addition, analogies between WHS-related epilepsy and Dravet syndrome (OMIM # 607208; a rare early-onset epilepsy syndrome characterized by refractory epilepsy and neurodevelopmental problems beginning in infancy) have led to speculation about the potential utility of newer agents used in Dravet syndrome (e.g., cannabidiol, stiripentol, fenfluramine) in drug-resistant WHS cases [12,13,14,16].

To date, most published research on WHS has focused on clinical and epidemiological descriptions, genetic findings, and genotype–phenotype correlations [1,2,3,4,5,6,10]. In contrast, detailed studies of epilepsy management in WHS remain scarce. Particularly, lacking is information regarding the use and effectiveness of newer antiseizure medications in this population. This study aims to provide a comprehensive description of epilepsy in a large cohort of 140 patients with WHS from Spain and Latin America. We compare our findings with the existing literature and explore the potential role of novel ASMs in improving clinical outcomes in this vulnerable population.

## 2. Results

### 2.1. Seizures: Descriptive Analysis of the Cohort

We report seizures on a cohort of 140 patients with a confirmed diagnosis of Wolf–Hirschhorn syndrome (WHS). Of the 137 patients for whom epilepsy data were available, 126 (92%) had experienced seizures at some point. At the time of the most recent data update, the median age of the cohort was 5 years (range: prenatal diagnosis to 13.2 years). The median age at diagnosis of WHS was 11 months (range: prenatal diagnosis to approximately 32 years). The mean age at seizure onset was 9.8 months (range: 3 days to 36 months) with a predominance of female patients (67.9%; 95/140); the ratio of female/male was 2.11/1. Additional descript data for the cohort is shown previously in Nevado et al. (2025) [10].

The main clinical features of the seizures are summarized in Table 2. Notably, most patients experienced more than one seizure type, with a frequent association with febrile contexts (in addition to non-febrile seizures), and a high incidence of status epilepticus was observed (Table 2).

Regarding the use of antiseizure medications (ASMs), 85.9% of patients with epilepsy were receiving ASM treatment at the time of the last data collection. Among them, fewer than half of them (47.4%) were on monotherapy. Most patients had experimented more than two different ASMs, and in 42.2% of cases, three or more ASMs had been used. Furthermore, 17.2% of children had received three or more ASMs concurrently at some point. The most commonly used drugs were valproic acid and levetiracetam, as shown in Table 3.

The current clinical status in terms of epilepsy control at the time of the last update is also summarized in Table 4. Interestingly, more than 50% of individuals were free of seizures for more than one year.

### 2.2. Subpopulation Analysis: Comparison Between the Two Sub-Cohorts

The study initially explored potential differences between Latin American and Spanish subpopulations regarding different epilepsy-related clinical items. Bi-variate correlations (Spearman and Pearson, as appropriate), Chi-square tests, and frequency data analysis were performed.

Despite some significant differences in some items, most clinical variables were similar between the two subpopulations (see Supplementary Materials), but lower overall epilepsy burden among the Spaniards was denoted. Consequently, as we assumed no major statistical differences between the two populations, the dataset was analyzed as a whole, although disaggregated data are available in Supplementary Materials.

### 2.3. Status Epilepticus in the Cohort

One of the most notable clinical observations in our cohort regarding epilepsy was the high frequency of status epilepticus observed, particularly during early infancy. This was consistent with previous reports (review by [12], and [13,14]). Bivariate correlation analyses using Pearson and Spearman coefficients to examine associations was also conducted, mostly between having status epilepticus (called “*status*”) and being admitted in Intensive Care Unit-status epilepticus (called “*ER-status*”, see Supplementary Materials) versus other clinical, developmental, and treatment-related variables of epilepsy.

Statistical analyses revealed that the “*status*” condition was significantly associated with multiple adverse clinical outcomes. In fact, Pearson correlations demonstrated positive associations with overall seizure presence (as expected; r = 0.322, *p* < 0.0001) but particularly with non-febrile seizures (r = 0.277, *p* = 0.001) as well as with specific seizure types, including generalized tonic–clonic (r = 0.191, *p* = 0.027), focal (r = 0.189, *p* = 0.029), and absence/atypical absence seizures (r = 0.293, *p* = 0.001). Furthermore, a strong positive correlation was also observed with several antiseizure medications (r = 0.445, *p* < 0.0001), especially valproic acid, clobazam/clonazepam, topiramate, and “*other ASMs*” (normally phenytoin, phenobarbital; r = −0.349, −0.250, −0.194, −0,362, respectively; *p* < 0.05). The “*status*” condition was also positively associated with comorbidities such as respiratory infections (r = 0.189, *p* < 0.05) and MRI-detected brain malformations (r = 0.200, *p* < 0.05).

Conversely, “*status*” was negatively correlated with ASM discontinuation (r = −0.060, *p* = 0.017) and key developmental milestones, including sitting unaided (r = −0.191), walking with and without support (r = −0.293, and r = −0.269, respectively), and word production (r = −0.199). Spearman correlations further supported these findings, showing significant associations between “*status*” with “*deletion size*” (r = 0.289, *p* = 0.002), “*Global Functional Assessment of the Patient*” score (GFAP, see [11] and Materials and Methods) (r = 0.508, *p* < 0.0001), “*number of status episodes*” (r = 0.885, *p* < 0.0001), “*number of total ASMs used*” (r = 0.445, *p* < 0.0001), and “*maximum concurrent ASMs*” (r = 0.439, *p* = 0.003). Negative correlations were also observed with “*seizure control*” (r = −0.257, *p* = 0.003) and “*motor development*” (r = −0.257, *p* = 0.003), indicating that to have status is in fact a marker of greater disease severity and developmental impact in those individuals. Similar results were also observed for the variable “*ER-status*” (see Supplementary Materials) as a marker of a more severe status complications.

In addition, having more episodes of status epilepticus was significantly linked to adverse clinical and functional outcomes. Specifically, a higher number of episodes was associated with a lower likelihood of stopping “*anti-seizure medications use*” (r = −0.207, *p* = 0.018), reduced “*ability to walk either with help*” (r = −0.236, *p* = 0.006) or “*unaided*” (r = −0.236, *p* = 0.006), poorer “*seizure control*” (r = −0.228, *p* = 0.008), and weaker “expressive language skills” (r = –0.179, *p* = 0.038). It was also linked to greater “*motor delay*” (r = −0.207, *p* = 0.016), and there was a clear trend toward more severe “*cognitive delays*” (data upon request).

Interestingly, a higher number of status epilepticus episodes was strongly and negatively associated with overall functional ability, as measured by the “*GFAP*” score (Nevado et al., 2025 [10]; r = −0.541, *p* < 0.0001). It was also related to worse developmental outcomes, including greater age-adjusted developmental milestones (r = −0.253, *p* = 0.003) and more frequent documented developmental impairments (r = −0.305, *p* < 0.0001).

Coversely, more status episodes were positively correlated with the presence of certain comorbidities. These included “*visual problems*” (r = 0.187, *p* = 0.03), repeated “*respiratory infections*” (r = 0.221, *p* = 0.01), “*brain malformations seen on MRI*” (r = 0.198, *p* = 0.023), and increased caregiver burden, such as situations where a family member “*had to leave their job to provide full-time care*” (r = 0.193, *p* = 0.025).

### 2.4. Ward’s Cluster Analysis

An unsupervised Ward’s cluster (Hastie et al., 2001) [17] analysis was conducted using the GFAP intermediate variable named “*global epilepsy*” (Table 5) as well as the whole GFAP (see Table S1 of Supplementary Materials). “*Global epilepsy*” was integrating over 12 different epilepsy features extracted from the questionnaires. Thus, four distinct clinical clusters emerged. The Z-tests for proportions revealed significant differences among clusters (Table 5).

#### 2.4.1. Status Epilepticus and Epilepsy-Related Severity

Cluster 4 exhibited the lowest severity, with a significantly reduced proportion of status epilepticus episodes compared to Clusters 1, 2, and 3 (*p* < 0.001) and fewer ICU admissions (*p* < 0.01). Cluster 3 showed an intermediate severity, with significantly fewer status cases than Clusters 1 and 2 (*p* < 0.001 and *p* < 0.01, respectively) but more than Cluster 4. In contrast, Clusters 1 and 2 represented the most severe clinical phenotypes, with the highest rates of status epilepticus and ICU admissions; no significant differences were observed between the last two clusters. These findings support the utility of the “*global epilepsy*” composite in stratifying clinical-epilepsy severity within the cohort.

#### 2.4.2. Epilepsy Severity and Deletion Size

**Figure 1:**
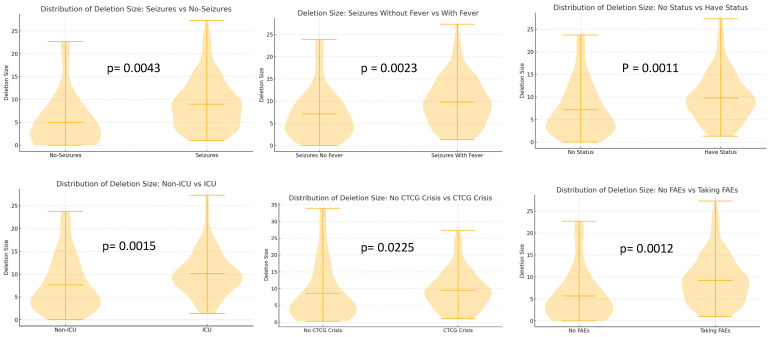
A comparison between the deletion size and different epileptic items. The Mann–Whitney U test to compare deletion sizes between patients who had the condition and those who did not was performed. Only differences that reached statistical significance were considered for further analysis. CTCG = GTCS, generalized tonic–clonic seizures; ICU, intensive care unit; FAES = AEDs.

Ward’s unsupervised cluster analyses based on the “global epilepsy” score also allowed us to investigate whether individuals with larger deletions and consequently with higher GFAP scores and worse prognosis are more likely to exhibit a severe or pharmaco-resistant epilepsy (Table 5). Detailed interpretation of the most striking results from the Table 5 highlights a clear gradient of epilepsy severity among clusters, with Clusters 1 and 2 showing the most significant burden of disease. Cluster 3 is intermediate in severity, with moderate seizure control, fewer status episodes, and lower GFAP levels. Finally, Cluster 4 was the least severe, characterized by late seizure onset, a predominance of febrile seizures, and the best seizure control. Although epilepsy severity appears clearly related to deletion size, as shown in comparisons between patients with small and large deletions, the relationship is not linear; rather, severity increases up to a certain threshold of deletion size, beyond which no further worsening is consistently observed. We additionally investigate whether deletion-size correlates with specific epilepsy-related items, comparing median deletion sizes between patients with and without a specific clinical condition. A summary of deletion size by different epilepsy-related items is shown in Figure 1.

Data extracted from the violin plot (shown above) illustrates that in the patients with seizures (mainly without fever) who had status, which may have ICU management, the deletion sizes were shifted toward larger values, and the statistical test (Mann–Whitney U test) confirms that this difference is statistically significant (Figure 1). This pattern supports the hypothesis that increased epilepsy severity may be linked to larger deletion sizes but is not strictly lineal.

Our comparisons (from violin plots and statistical tests) may also estimate putative critical regions associated with refractory epilepsy-related phenotypes in patients within the Wolf–Hirschhorn syndrome region. These regions are based on the average deletion size in patients presenting each clinical condition, assuming the deletion extends from the telomeric end of chromosome-4p. Thus, larger deletions (median up to ~10 Mb) tend to present more “severe” or treatment-resistant epilepsy, potentially linked to genes located there.

##### Genes and Treatment-Resistant Epilepsy

In our cohort of 140 individuals with WHS, we have observed that epilepsy is highly heterogeneous and often showed refractory features. While the haploinsufficiency of genes located near the telomere, such as *PIGG*, *CPLX1*, *CTBP1*, and *LETM1*, has been traditionally implicated in seizure susceptibility [6,8,9], our data suggest that the most severe, drug-resistant forms of epilepsy may be associated with larger deletions extending proximally to around 8–11 Mb on chromosome-4p. Interestingly, this interval encompasses the dopamine receptor D5 gene (*DRD5*), whose loss could impair dopaminergic modulation of cortical and hippocampal excitability [18,19].

When comparing individuals with *DRD5*-inclusive deletions ≥ 9.5 Mb versus those with smaller deletions (<9.5Mb; *DRD5+*; Table 6), we observed that *DRD5’s deleted* group showed an earlier seizure onset (8.21 ± 4.70 vs. 10.85 ± 7.53 months, *p* = 0.013) and exhibited a higher frequency of status epilepticus (68.4% vs. 50.6%, *p* = 0.037; OR = 2.20). They also required a higher maximum number of antiepileptic drugs simultaneously used (1.74 ± 0.95 vs. 1.49 ± 0.89, *p* = 0.013) and demonstrated worse seizure control (2.85 ± 0.48 vs. 4.14 ± 1.50, *p* = 2.63 × 10^−17^). Moreover, motor and cognitive development were significantly more affected in the large-deletion group (*p* < 0.001 for both), accompanied by higher global comorbidity burden (*p* = 0.0029) and markedly increased of GFAP scores (260.76 ± 62.25 vs. 205.90 ± 75.50, *p* = 8.94 × 10^−6^). Finally, these patients were diagnosed earlier than *DRD5+* individuals (12.41 vs. 37.01 months, *p* = 0.016), with a worse seizure pattern (see Table 6).

**Figure 2:**
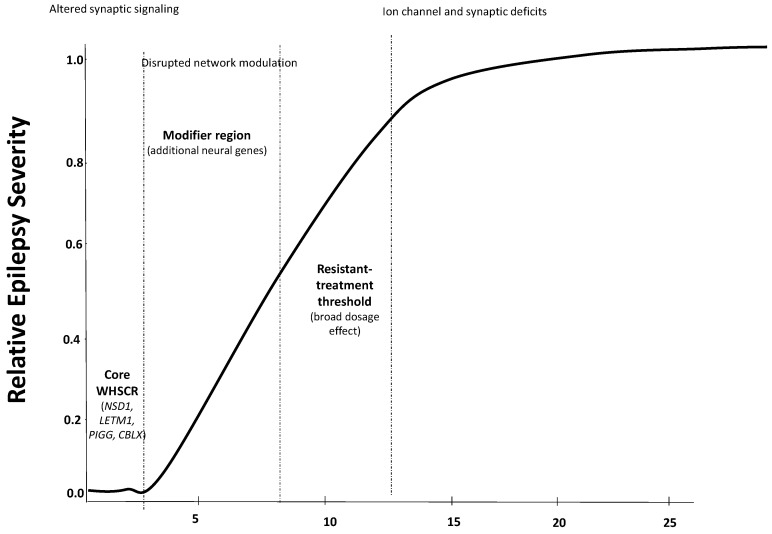
Integrated model of hypothetized epileptogenesis in Wolf–Hirschhorn syndrome.

Thus, treatment-resistant epilepsy line up with larger median deletion sizes, consistently in the ~8–11 Mb range (from the telomeric end of chromosome-4p), compared with ~5 Mb for milder cases. Altogether, let us show putative integrated model of complex epileptogenesis in WHS (Figure 2).

#### 2.4.3. Age and Status Epilepticus

To examine whether or not age may be associated with the presence or severity of status epilepticus, we performed Ward’s cluster analysis using the variable “*age at evaluation*” as the defining variable, yielding three age-clustered based groups (see Table S1 of Supplementary Materials). Cluster 1 (mean age 32.11 years; n = 8) included older individuals, Cluster 2 (mean age 16.00 years; n = 24) had mainly adolescents, and Cluster 3 (mean age 4.21 years; n = 107) represented the younger or the pediatric group. While the adolescent group had the highest proportion of patients with status epilepticus (66.7%), the differences among clusters were not statistically significant. Even when combining Clusters 1 and 2 (older patients), the proportion of individuals with *status* (62.5%) was only slightly higher than that observed in the pediatric group (55.3%), showing a non-significant statistical trend to (*p* > 0.05).

### 2.5. Epilepsy and Comorbidities in WHS?

Epilepsy in patients with WHS frequently coexists with an important number of comorbidities, including psychomotor developmental delay, hypotonia, and behavioral issues, among others. As we expected, a significant correlation was found between the presence of seizures and renal/urological anomalies (r = 0.208, *p* = 0.015), suggesting a notable link between seizure occurrence and kidney or urinary tract involvement. In addition, in patients with non-febrile seizures, the brain malformations (denoted by MRI) were also significantly associated with epilepsy (r = 0.182, *p* = 0.015), indicating that structural brain abnormalities may be associate with seizure susceptibility or vice versa. Furthermore, the overall burden of comorbidities, as quantified by the GFAP, was positively correlated with seizures (r = 0.178, *p* = 0.038) and more specifically with non-febrile ones (r = 0.206, *p* = 0.017). These findings may emphasize that the presence and severity of epilepsy in WHS are influenced by the complexity of comorbid conditions. The cluster analysis further clarified the interplay between epilepsy and comorbidities, revealing distinct clinical subgroups among patients (see Supplementary Materials).

In addition, Ward’s analysis displayed that Cluster 1 showed the most severe epilepsy phenotype and poor seizure control and also had the highest burden of comorbidities, including nephro-urological and cardiovascular anomalies as well as a greater frequency of surgical interventions, aligning with its overall severe clinical presentation. In contrast, Cluster 4 represented the mildest profile, with the best seizure control, lowest epilepsy severity, and minimal comorbid burden. Clusters 2 and 3 showed intermediate levels of severity, with Cluster 3 notably enriched in MRI-detected brain malformations and Cluster 2 presenting the highest rate of recurrent infections. Taken together, these findings suggest that certain comorbidities, but not all, may contribute to a worsening of the overall epilepsy phenotype. Further analysis would be required to determine which specific comorbidities play the most significant role in modulating seizure burden across WHS subgroups.

### 2.6. Age and Epilepsy Evolution Management?

Although epilepsy in WHS is often drug-resistant, many patients show improved seizure control with age, particularly during adolescence [4]. Our analysis revealed no significant differences in age across clinical severity clusters, suggesting that age at evaluation does not strongly influence epilepsy phenotype (see above). However, an individual with older age was positively associated with being off antiseizure medications (ASMs) (r = 0.254, *p* = 0.003), indicating that older patients were more likely to have a discontinued treatment, as it has been previously suggested.

Conversely and interestingly, the monotherapy was not directly linked to ASM age-based discontinuation but was inversely associated with several markers of clinical severity, such as *failure to thrive* (r = −0.224, *p* = 0.009), *non-febrile seizures* (r = −0.255, *p* = 0.003), *history and higher number of status epilepticus episodes* (r = −0.206, *p* = 0.017, and r = −0.204, *p* = 0.018, respectively), *and caregiver job cessation* (r = −0.186, *p* = 0.031). A weak, non-significant trend also suggested that non-febrile seizures may decrease with age (r = −0.161, *p* = 0.060). Overall, the findings hint at a possible improvement in epilepsy over time, although the young age of most patients in our cohort may have limited the detection of stronger age-related patterns.

### 2.7. Fever Sensitivity and Antiseizure Medication Response?

Febrile-triggered seizures are frequent in young WHS individuals [12], particularly in those with generalized epilepsy phenotypes. In this clinical context, some reports described poor seizure control or even paradoxical worsening with sodium channel-blocking antiseizure medications (carbamazepine, oxcarbazepine, and phenytoin), warranting caution in their use [12,14]. However, to date, no direct mechanistic association between fever (as a trigger of epilepsy) and these drugs has been established. In our cohort, no specific ASM showed any significant association with febrile seizures, including those previously thought to be contraindicated (data upon request), although we observed a positive correlation between having “*febrile seizures*” and the “*current use of any ASM*” (r = 0.306, *p* < 0.0001), as we expected. In contrast, we established stronger associations between having non-febrile (other causes beside fever) seizures and different ASM-treatments. These correlations included higher overall “*ASM use*” (r = 0.365, *p* < 0.0001); the use of “*valproate*” (r = 0.206, *p* = 0.017) or, negatively, with “*diazepam*” (r = −0.205, *p* = 0.017); and “*other ASM*” categories (normally, phenitoin, and phenobarbital; r = −0.222, *p* = 0.010).

## 3. Discussion

Wolf–Hirschhorn syndrome is associated with a high incidence of early-onset epilepsy. The prevalence of epilepsy in our cohort (92%), along with hallmark features such as an early onset (typically before one year of age) and a tendency for seizure worsening during febrile episodes, is consistent with previous reports [12,13,14]. We also highlighted a marked seizure aggressiveness during infancy, which is reflected in the high rate of status epilepticus observed in our cohort (58.4% of our patients experienced at least one episode of status epilepticus), a figure slightly higher than ~50% reported in previous studies [12,14]. This may be partly attributed to updated International League Against Epilepsy (ILAE) criteria, which define status with shorter duration thresholds. Regardless, the consistency of findings across geographic regions is striking: a substantial proportion of WHS patients worldwide exhibit recurrent status epilepticus and require multiple antiseizure medications (ASMs) to achieve partial or full seizure control.

Table 7 summarizes our results in comparison with those of the main studies published to date (September 2025). Overall, no major differences in epilepsy characteristics are evident across cohorts, with similar profiles observed in terms of seizure types and ASM use patterns.

As revised above, a hallmark of epilepsy in WHS is the high incidence of status epilepticus, particularly in early childhood. Our findings align with prior results reviewed by Paprocka et al., 2022 [12], who noted a similar frequency of status epilepticus and emphasized its concentration in the early years of life as well as its association with more severe developmental outcomes. Ho et al., 2018 [14], also acknowledged status epilepticus as a common feature among patients with severe seizure phenotypes, incorporating it as a key criterion in their gene prioritization framework. Our study supports and extends these observations and demonstrates that status epilepticus is closely linked to increased seizure burden, more complex seizure types, higher pharmacological load, and poorer developmental outcomes in patients with WHS. In fact, notably, stronger statistical associations were found with (i) delayed motor and cognitive milestones; (ii) a higher number and complexity of ASMs; and (iii) specific comorbidities such as respiratory infections, hearing abnormalities, and feeding difficulties. Conversely, the significant inverse correlations between the existence of status and seizure control reinforce the clinical relevance of status events as potential markers of epilepsy refractoriness. Thus, experiencing status epilepticus and having a higher number of status episodes are all statistically and significantly associated with poorer functional outcomes, developmental impairment, and increased comorbidity. Our data also suggest that the occurrence and frequency of status epilepticus in WHS are not strictly age dependent. Although there are mild trends toward increased severity in adolescents and older individuals, these differences do not reach statistical significance.

Importantly, our data also confirm previous descriptive reports suggesting that a higher number of status epilepticus episodes correlates with more pronounced psychomotor delay [4,5,6,12]. This reinforces the idea that early and frequent epileptic decompensations may have a cumulative detrimental effect on neurodevelopment. Interestingly, at least half of individuals (in our cohort) experienced highly aggressive epilepsy during the early stages of neurodevelopment, frequently marked by recurrent seizures and episodes of status epilepticus. These findings underscore the importance of optimizing epilepsy management in early childhood, with a focus on antiseizure medications that offer both efficacy and a favorable cognitive side-effect profile.

Clinicians may have internalized, in some cases, the idea that epilepsy severity in WHS is a transient phase, inadvertently adopting a therapeutic tolerance in anticipation of spontaneous improvement. However, considering the increasingly broad arsenal of ASMs now available in many healthcare settings, such as cannabidiol or flenfluramine, which are indicated for Dravet syndrome (a condition in which epilepsy shares many characteristics with and that has been seen in WHS), such a passive approach is no longer tenable. Thus, early, proactive, and individualized intervention is essential to minimizing long-term neurological harm and improving outcomes for patients with WHS.

Regarding seizure types, nearly all patients experienced more than one, with generalized tonic–clonic seizures (GTCS) and atypical absence seizures being the most common. These findings are consistent with prior studies (reviewed by Paprocka et al., 2022 [12]) and support the notion that seizure generalization and variability are hallmarks of the WHS epilepsy phenotype. The use of ASMs also reflected global practice, with valproic acid (VPA) and levetiracetam (LEV) as first-line agents and widespread use of benzodiazepines (CLB/CLZ, DZP) for both maintenance and acute rescue. Phenobarbital still remained in use, particularly in the Latin American subset, despite the profile of adverse effects, especially in terms of cognitive function. Lacosamide was consistently reported as ineffective or harmful, aligning with previous findings that suggest sodium channel-targeting ASMs may be less effective or contraindicated in this population.

Our data also trend a non-strict linear relationship between deletion size and the epilepsy severity. In fact, these suggest that while the minimal critical region for Wolf–Hirschhorn involves ~0.5–2 Mb (from the *p*-telomere), extended deletions larger than 5–6 Mb were more likely starting to be associated with difficult-to-control epilepsy. Beyond ~8–10 Mb (encompassing additional neural genes) underlie the most severe, refractory forms of epilepsy. Interestingly, this interval encompasses the dopamine receptor D5 gene (*DRD5*), whose loss could impair dopaminergic modulation of cortical and hippocampal excitability. The DRD5 receptor belongs to the D1-like receptor family, which activates cAMP–PKA signaling and enhances excitatory neurotransmission [18,19]. The disrupting effect of DRD5 signaling could therefore tilt the excitation–inhibition balance toward hyper-excitability, modulating the counter-balancing effect between D1- and D2-like receptors in WHS individuals. This is consistent with the emerging recognition of the dopaminergic control in cortical excitability and epileptogenesis [20] and warrants further validation through integrative genomic, transcriptomic, and functional neuroimaging studies assessing dopaminergic pathway integrity in WHS individuals.

Investigating regions beyond the so-called “critical syndrome region” could help better understand epilepsy in these patients. Indeed, “refractory indicators, such as status epilepticus (and higher number of them), a high seizure burden, the use of a higher number of ASMs, and ICU management, are presented in individuals, which all clustered at median deletions over 9 Mb. Thus, this also may suggest a three-tier model of epileptogenesis in WHS: (1) a “core” epileptogenic region within the first 0–2 Mb that initiates seizures, (2) a modifier region within 2–8 Mb, and (3) extended deletions beyond ~8–11 Mb that involve additional dosage-sensitive genes and drive a treatment-resistant, severe epilepsy phenotype (see Figure 2). This integrated suggested model could explain how deletion size governs epilepsy severity in Wolf–Hirschhorn syndrome. A core WHSCR (0–2 Mb) containing the following genes, *NSD1*, *LETM1*, *PIGG*, *CPLX1*, and *CBTP1*, may be synergistically sufficient to trigger seizures but generally are responsive to treatment. A modifier region (2–8 Mb) encompasses additional neural-expressed genes that incrementally worsen the epileptic phenotype. Finally, there is a resistant-epilepsy threshold (>8–11 Mb), where loss of broad dosage-sensitive genes leads to a steep rise in treatment-resistant epilepsy.

The logistic curve denotes relative epilepsy severity climbing sharply once the deletion extends past ~8 Mb (Figure 2). This framework provides a quantitative, mechanistic model explaining why larger 4p-deletions may correlate with the most severe, refractory epilepsy in this syndrome. Thus, deletions over 12 Mb do not seem to contribute more to this epilepsy complexity. Nevertheless, not all large deletions resulted in severe phenotypes, nor were all small deletions benign. This may suggest that specific genetic content, rather than deletion size alone, could also be crucial. These findings echo the multigenic and potentially epigenetic models (a fact that we are checking) proposed by Ho et al., 2018 [14], and highlight the need for functional analyses of candidate genes beyond the classical WHS critical region.

The accepted concept that epilepsy improves with age in WHS was also partially supported by our data. In fact, older age individuals (at evaluation time) correlated significantly with an ASM discontinuation but not with seizure freedom or significant clinical improvement and were observed at most of the clusters analyzed. This indicates that while improvement is possible, especially beyond adolescence, early and severe epilepsy could leave a lasting neurodevelopmental impact. Thus, early intervention remains critical. Interestingly, non-febrile-related seizures emerged as a potential clinical marker of severity in our cohort. These were significantly associated with poly-therapy, more status episodes, larger deletions, and worse functional outcomes. While Paprocka and colleagues [12] and Ho et al. [14] did not explore this association explicitly, their descriptions of severe phenotypes often included atypical absences and tonic seizures. Our findings extend this observation and suggest that afebrile seizure onset may serve as an early predictor of a potential refractory epilepsy.

On the other hand, previous studies have suggested that febrile-triggered seizures may characterize a specific epilepsy phenotype within WHS. In this context, some reports describe suboptimal seizure control or even paradoxical worsening with sodium channel-blocking antiseizure medications (ASMs), particularly carbamazepine, oxcarbazepine, and phenytoin [12,14]. Paprocka et al. (2022) [12] noted that febrile episodes frequently precipitate seizures or status epilepticus in young WHS children, while Ho et al. (2018) [14] highlighted pharmaco-resistance to these agents in patients with generalized epileptic phenotypes, drawing parallels with Dravet-like profiles. However, no link between the two facts has been reported so far. Thus, we explored this situation, but we did not observe association between febrile-related seizures and resistance to any specific ASM. Instead, non-febrile seizures were more strongly linked to worse epilepsy outcomes. Therefore, although caution remains warranted when considering sodium channel-modulating ASMs in WHS, fever sensitivity alone does not appear to be a reliable indicator of treatment resistance in our series. Rather, the presence of non-febrile seizures (alone or with fever ones) may better identify individuals with more severe or treatment-refractory epilepsy requiring complex therapeutic strategies.

Finally, our analysis highlights the critical role of comorbidities in epilepsy management. Patients with more severe seizures more frequently exhibited multisystem involvement, and comorbidities such as respiratory, renal, and hearing issues were associated with increased status epilepticus and poorer seizure control. These comorbid conditions complicate epilepsy management and underscore the need for a comprehensive, individualized approach to treatment, one that takes into account the type of seizures and the broader clinical profile, including the selection of ASMs with the most appropriate safety and efficacy profile for the comorbidities present.

In summary, this study offers one of the most comprehensive clinical evaluations to date of epilepsy in individuals with Wolf–Hirschhorn syndrome (WHS), with a robust cohort of 140 patients and detailed phenotyping. Our results confirm key features already described in the literature, such as an early onset, multiple seizure types, and a high rate of pharmaco-resistance, while also providing new insights into the clinical heterogeneity and therapeutic challenges of this population.

### Future Directions

**Figure 3:**
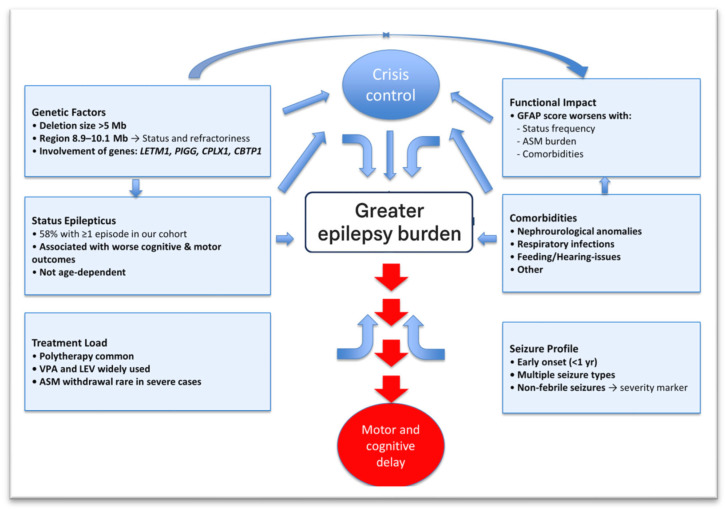
Critical factors influencing epilepsy severity in WHS.

As our understanding of Wolf–Hirschhorn syndrome (WHS) continues to evolve, we may witness a gradual shift toward a milder clinical phenotype, increasingly distinct from classical descriptions (see Figure 3). This transformation is likely driven by several converging factors: (i) The widespread adoption of advanced and increasingly accessible genetic diagnostic technologies has enabled the identification of smaller deletions, which are often associated with less severe clinical presentations. (ii) The earlier implementation of developmental surveillance and neurorehabilitation programs has allowed for therapeutic interventions to begin during critical periods of brain plasticity. (iii) The advent of a new generation of antiseizure medications (specifically developed for epileptic encephalopathies) offers improved efficacy and tolerability, potentially translating into better seizure control and, ultimately, enhanced quality of life and neurodevelopmental trajectories for individuals with WHS. Collectively, these trends highlight a transition from a historically severe and poorly understood condition to a more nuanced, manageable spectrum disorder. Continued research into genotype–phenotype correlations, treatment responsiveness, and longitudinal outcomes will be essential to refining clinical strategies and further improving care in WHS.

## 4. Materials and Methods

### 4.1. The Cohort

Data from patients with WHS were prospectively collected within the last 11 years (2013–2023). One hundred and forty (140) individuals with genetic diagnosis of WHS were finally recruited. Part of this cohort was previously included and partially described by us [4,6,10]. The cohort is constituted by subjects from different nationalities; the majority were recruited from Spain (n = 75; 2 of them with Morocco’s origin) and Latin America (n = 65), where different nationalities are represented: Argentinians (39) Mexicans (9), Peruvians (1), Chileans (5), Venezuelans (6), Ecuadorians (3), and Colombians (2). The clinical information of patients was obtained from the referring physicians and was revised by two independent geneticists, and two neuropediatricians using two standardized questionnaires completed with data of the medical reports, and interviewing most of the parents. The caregiver questionnaire proved to be reliable and valid, as evidenced by its successful use in over 150 patients, with no missing or misunderstood items reported by parents. Its validity was further confirmed through medical record verification by two geneticists and two neuropediatricians and by parent interviews conducted by two professionals with expertise in the syndrome. To the Latin American group, the questionnaire was administered in the same language (Spanish) and cultural context across all participants. No cultural adaptation process was required because the completion of the form was conducted directly with a geneticist during meetings held with patients and families at the Latin American support group gatherings in Buenos Aires (Argentina). A complete characterization of the cohort was available in Nevado et al., 2025 [10].

### 4.2. Methods

This study was based on data collection from patients diagnosed with Wolf–Hirschhorn syndrome (WHS) in Spain and Latin America between 2013 and 2023. The inclusion criterion was a confirmed genetic diagnosis of WHS established by microarray-based analysis, either using SNP arrays (Single Nucleotide Polymorphism arrays; CytoSNP850K v1.2 from Illumina, San Diego, CA, USA), mostly, or comparative genomic hybridization (CGH; KaryoArray™, [21]).

Data were collected in collaboration with the Spanish Wolf–Hirschhorn Syndrome Association (AESWH), which facilitated access to families and supported the dissemination of online self-administered questionnaires. These questionnaires were subsequently cross-referenced with clinical documentation provided by the families and submitted to the research team through the AESWH. In some cases, particularly during the initial phase of data collection, epidemiological and clinical forms were completed directly by the pediatric neurologists responsible for the care of each patient. Regarding epilepsy-specific information, caregivers were first educated on the clinical features associated with different seizure types before completing the relevant sections of the questionnaire. The accuracy of the reported seizure types and ASM use was corroborated using the patients’ clinical records. The same questionnaires were distributed to families in Latin America, also via AESWH. Most of the patients were personally evaluated (by JN) during the in-person meetings and family workshops organized annually in Buenos Aires (Argentina) from 2014 to 2019.

The data collection began with an initial cohort of 27 children and was progressively updated over the years, reaching up to 140 patients by the 2025 review [10]. In all cases, parents or legal guardians were informed about the nature and objectives of the study, and signed informed consent was obtained. Clinical data were anonymized to ensure confidentiality.

The GFAP (Global Functional Assessment of the Patient) score was constructed (using different variables from the questionnaires) as a continuous composite metric integrating four major clinical domains in the syndrome: (i) age-adjusted psychomotor milestones, (ii) epilepsy features, (iii) presence of comorbidities, and (iv) developmental aspects. Each item was scored based on clinical severity and weighted according to Human Phenotype ontology (HPO; https://hpo.jax.org/; accessed on 20 January 2022) term frequencies and clinical relevance. Detailed information regarding this score was previously described by us [10].

Statistical analyses were performed with SPSS version 29 (IBM Corporation, Chicago, IL, USA). Descriptive analysis included mean ± SD for continuous variables and frequency tables for categorical variables. The categorical variables were taken from our two questionnaires curated from medical records and were expressed as “1” (condition present at some point) or “0” (condition not present at any time). Correlation associations were calculated using Pearson’s linear correlation coefficient (continuous variables) or Spearman’s Rho and Kendall’s tau_b (categorical variables). Comparisons between the two groups were performed with either Student’s *t*-test (for continuous variables), Mann–Whitney U test, or Chi-square tests (for categorical ones). For more than two groups, ANOVA (followed by Bonferroni’s or T3- Dunnett post hoc tests) were run for continuous variables and z-tests between column proportions for categorical variables. Ward’s minimum variance method was the criterion used in hierarchical cluster analysis, and the number of clusters was selected using the Bayesian Information Criterion (BIC) or Akaike Information Criterion (AIC). A *p*-value lower than 0.05 was considered to indicate statistically significant differences. The results were compared with the published literature indexed on epilepsy in WHS to contextualize the findings.

## 5. Conclusions

- Epilepsy is a highly prevalent and clinically burdensome feature of WHS, typically beginning in early infancy and frequently characterized by multiple seizure types, pharmaco-resistance, and status epilepticus.
- A substantial proportion of patients, particularly in early childhood, experience severe and refractory epilepsy, significantly affecting neurodevelopment and family quality of life. Early, individualized, and aggressive therapeutic strategies are critical, with careful selection of ASMs that balance efficacy and cognitive tolerability.
- Epilepsy severity appears to correlate with chromosomal deletion size, particularly beyond 5 Mb and up to 11 Mb on 4p-ter arm, although this relationship is not strictly linear, suggesting additional genetic or epigenetic influences.
- Non-febrile-related seizures may serve as a clinical marker of epilepsy severity and treatment refractoriness in WHS.
- While some patients show improvement over time, age alone does not predict better outcomes. Proactive management in early life remains essential.
- Comorbidities are strongly associated with epilepsy severity and should guide therapeutic decisions. A multidisciplinary care model is essential for optimizing outcomes.
- Advances in early diagnosis, neurodevelopmental care, and access to newer-generation ASMs offer a pathway to milder clinical trajectories in WHS and should be integrated into standard care frameworks.

## Figures and Tables

**Table 1:** Clinical features of patients with Wolf–Hirschhorn Syndrome.

Clinical Feature	Frequency (%)	Comments
Psychomotor developmental delay	98.90	Typically evolves into moderate-to-severe intellectual disability; expressive language particularly affected
Epilepsy	98.90	Often presents with specific and recognizable features
Characteristic facial features	97.80	Most of individuals had
Hypotonia	89.0	A great number of individuals had at some point
IUGR	94.20	Despite adequate caloric intake
Congenital heart defects	44.50	Includes ASD, PS, VSD, PDA, and AI
Renal and urological anomalies	53.0	Renal agenesis or dysplasia, vesicoureteral reflux, cryptorchidism
Behavioral disorders, Autism spectrum disorder	22.0–62.8	Stereotypes, impulsivity
Dental abnormalities	50.0–66.0	Delayed tooth eruption, bruxism, micrognathia, tooth agenesis (especially oligodontia)
Other: Immunodeficiency, adult-onset tumor predisposition, midline defects (e.g., cleft lip/palate)	Variable	Depending on the source
Seizures triggered by febrile illness (outside epilepsy context)	68.6%	

ASD, atrial septal defect; PS, pulmonary stenosis; VSD, ventricular septal defect; PDA, patent ductus arteriosus; AI, aortic insufficiency. Table adapted from data of Nevado et al. (2025) [10] with the cohort studied herein.

**Table 2:** Types of seizures in the cohort (n = 137).

Seizure Type	Frequency (%)	Comments
GTCS	55.9	Many patients experienced more than one seizure type
AATS	51.8	
FS	26.9	
TS	24.3	
MS	20.4	
ES	12.4	
SE	58.4	Defined as seizures lasting ≥10 min; more than half of patients experienced at least one episode
Among patients who experienced status epilepticus:		
Subgroup	Frequency (%)	
Only one episode of SE	17.9	
More than one episode of SE	39.2	
More than five episodes of SE	17.9	Nearly 20% of patients with SE had five or more episodes
Other Findings	Frequency (%)	
Seizures triggered by febrile illness (outside epilepsy context)	68.6%	

Abbreviations: GTCS, generalized tonic–clonic seizures; AATS, absence or atypical absence seizures; FS, focal seizures; TS, tonic seizures; MS, myoclonic seizures; ES, epileptic spasms; SE, status epilepticus.

**Table 3:** Antiseizure medications used in the cohort.

ASM	Number of Patients (% of ASM-Treated Group)	Comments
VPA	84 (62.2%)	
LEV	49 (36.3%)	
CLB/CZP	29 (21.5%)	
LMT	15 (11.1%)	
OXC/CBZ	13 (9.6%)	
TPM	7 (5.2%)	
ETS	3 (2.2%)	
DZP	8 (5.9%)	Used intermittently during febrile illness; not part of regular ASM regimen
Other ASMs	42 (31.1%)	Mainly PB in infants, especially in Latin America; some LCM) and BRV

VPA, Valproic acid; LEV, Levitaracetam; CLB/CZP, Clobazam/Clonazepam; LMT, Lamotrigine; OXC/CBZ, Oxcarbazepine/Carbamazepine; TPM, Topiramate; ETS, Ethosuximide; DZP, Diazepam; ASM, antiseizure medication; PB, phenobarbital; LCM, lacosamide; BRV, brivaracetam. n = 135 patients receiving ASM treatment.

**Table 4:** Seizure control status in the WHS cohort (n = 137).

Level of Control of Crisis	Seizure Control Level	Frequency (%)
1	Ongoing monthly seizures	19/126 (15.07)
2	Less than 3 months seizure-free	20/126 (15.87)
3	Seizure-free for 3–12 months	23/126 (18.25)
4	Seizure-free for 1–2 years	14/126 (11.11)
5	Seizure-free for 2 years or more	50/126 (39.68)
6	Never had seizures *	11/137 (8.29)

* Non-reported seizures by medical specialists at any time.

**Table 5:** Ward’s clusters based on “*global epilepsy*” variable (n = 137).

“*Global Epilepsy*”	Cluster 4	Cluster 3	Cluster 2	Cluster 1
GFAP (score)	108.24 ± 20.57 (113) * range 64–136	218.69 ± 50.16 (213) ** range 94–347	277.74 ± 41.61 (274) *** range 210–369	329.43 ± 48.61 (330) range 250–410
Size of deletion (Mb)	4.79 ± 4.45 (3.44) * range 0.01–10.73	8.67 ± 5.36 (8.88) ** range 1.30–23.73	11.15 ± 6.18 (9.87) range 1.77–21.29	9.86 ± 3.36 (9.78) range 3.94–15.65
To have Seizures	21/31 (67.74%) *	55/56 (98.21%)	38/38 (100%)	13/13 (100%)
Crisis control (1–6) ^&^	5.20 ± 0.80 (5) * range 3–6	3.67 ± 1.56 (4) range 1–5	2.86 ± 1.18 (3) range 1–5	2.08 ± 1.44 (2) range 1–5
Age of seizures (months)	10.74 ± 10.62 (11) range 2–36	10.91 ± 5.87 (10) range 2–30	8.914 ± 3.51 (8) range 1–18	7.83 ± 4.23 (9) range 0.01–12
Seizures w fever	18/31 (58.06%) *	41/56 (73.21%) ***	26/38 (68.42%) ***	11/13 (84.61%)
Seizures w/o fever	3/31 (9.67%) *	32/56 (55.00%) **	33/38 (94.73%)	13/13 (100%)
Status	3/31 (9.67%) *	29/56 (51.78%) **	36/38 (86.79%)	12/13 (76.19%)
Number status	0.58 ± 2.21 (0) * range 0–11	1.43 ± 3.08 (1)** range 0–20	3.97 ± 3.41 (3) *** range 0–13	12.30 ± 15.93 (5) range 0–55
Status to ICU	2/31 (6.45%) *	18/56 (32.14%) **	28/38 (73.68%)	10/13 (76.92%)
Take ASM, now	13/31 (41.94%) *	52/56 (92.86%)	38/38 (100%)	13/13 (100%)
Number of ASM	1.03 ± 1.23 (1) * range 0–5	1.91 ± 1.11 (2) ** range 0–4	2.89 ± 1.20 (3) range 1–6	3.53 ± 1.50 (3) range 1–6
	1.03 ± 1.23 (1) * range 0–5	1.91 ± 1.11 (2) ** range 0–4	2.89 ± 1.20 (3) range 1–6	3.53 ± 1.50 (3) range 1–6
Monotherapy	12/31 (30.00%) ^$^	34/56 (65.00%) **	15/38 (45.28%) ***	3/13 (33.33%)
Max. (#) ASM used simult.	0.72 ± 0.70 (1) * range 0–2	1.54 ± 0.74 (1) ** range 0–3	2.07 ± 0.81 (2) range 1–5	2.30 ± 0.85 (2) range 2–4
Took ASM, not now	11/31 (38.70%) *	9/56 (16.07%) **	0/38 (0%)	0/13 (0%)

ASM, antiseizure medication; GFAP, global functional assessement of the patient; ICU, intentive care unit; (^&^) see Table 4 for explanation; #, number; (*) *p* < 0.05 vs. CL3, CL2, CL1; (**) *p* < 0.05 vs. CL2, CL1; (***) *p* < 0.05 vs. CL1; (^$^) *p* < 0.05 vs. CL3, CL2.

**Table 6:** Clinical and epilepsy-related features in individuals with WHS stratified by *DRD5* deletion status.

Variable	*DRD5* (Deleted) ≥9.5 Mb	*DRD5* (Not Deleted) <9.5 Mb	*p*-Value
Deletion size	15.04 ± 6.48 *	5.05 ± 2.85	*p* = 6.50 × 10^−17^
Sub-population	33/24	42/41	*p* = 0.50
Age evaluation (years)	7.07	9.82	*p* = 0.189
Age diagnosis (months)	12.41 *	37.01	*p* = 0.016
gender (male/female)	22/35 (1:1.6)	23/60 (1:2.6)	*p* = 0.241
seizures	52/57 (91.22%)	74/83 (85.15%)	*p* = 0.189
Age seizures onset (months)	8.21 ± 4.70 *	10.85 ± 7.53	*p* = 0.013
Seizures with fever	34/57 (63.15%)	60/83 (72.29%)	*p* = 0.17
Seizures no fever	39/57 (68.42%) ^t^	43/83 (51.80%)	*p*-value (Fisher exact test) = 0.056; OR = 2.02
SE	39/57 (68.42%) *	42/83 (50.60%)	*p*-value (Fisher exact test) = 0.037; OR = 2.20
Number of Status	3.01	3.03	*p* = 0.99
ICU Status	30/57 (49.12%) ^t^	29/83 (34.94%)	*p*-value (Fisher exact test) = 0.055; OR = 2.07
ASMs	50/54 (92.60%)	66/80 (82.5%)	*p* = 0.30
Number of ASMs	2.22 ± 1.34	2.11 ± 1.50	*p* = 0.65
Max. ASMs simultaneously	1.74 ± 0.95 *	1.49 ± 0.89	*p* = 0.013
Other ASMs	23/57 (40.35%) ^t^	20/83 (24.09%)	*p*-value (Fisher exact test) = 0.062; OR = 2.13
Took ASMs not now	6/57 (10.52%)	14/83 (16.86%)	*p* = 0.42
Crisis control	2.85 ± 0.48 *	4.14 ± 1.50	*p* = 2.63 × 10^−17^
Cardiovascular alterations	26/57 (45.61%)	35/83 (42.16%)	*p* = 0.82
Nefruourological alterations	36/57 (63.15%) *	37/83 (44.57%)	*p*-value (Fisher exact test) = 0.039; OR = 2.13
Ophthalmology alterations	37/57 (64.91%) ^t^	42/83 (49.39%)	*p*-value (Fisher exact test) = 0.056; OR = 2.05
Malformation CNS after MRI	32/42 (76.19%)	41/58 (70.69%)	*p* = 0.54
Surgeries	1.22 ± 1.43	0.85 ± 1.15	*p* = 0.11
Familiar quit job	38/57 (64.91%) ^t^	41/83 (51.80%)	*p*-value (Fisher exact test) = 0.055; OR = 2.07
Motor delay	2.11 ± 1.77 *	4.20 ± 1.73	*p* = 5.46 × 10^−10^
Cogniteve delay	2.09 ± 1.07 *	2.91 ± 1.33	*p* = 0.00011
Developmental delay corrected by age	27.53 ± 10.00 *	16.21 ± 10.84	*p* = 6.22 × 10^−09^
Comorbidities	9.46 ± 4.58 *	7.10 ± 4.12	*p* = 0.0029
Alterations affecting developmental	143.50 ± 39.72 *	121.75 ± 43.47	*p* = 0.00312
Global epilepsy	80.43 ± 27.56 *	59.89 ± 31.06	*p* = 0.000090
GFAP score	260.76 ± 62.25 *	205.90 ± 75.50	*p* = 8.94 × 10^−06^
GTCS	37/57 (64.91%) *	39/83 (46.99%)	*p*-value (Fisher exact test) = 0.040; OR = 2.13
AATS	25/57 (64.91%)	46/83 (48.19%)	*p* = 0.19
MS	14/47 (24.56%)	14/83 (18.86%)	*p* = 0.35
PS	19/57 (33.33%)	17/83 (20.48%)	*p* = 0.092
ES	6/57 (10.52%)	11/83 (13.25%)	*p* = 0.65
TS	11/57 (19.29%)	23/87 (26.43%)	*p* = 0.27
Mortality	6/57 (10.5%) ^t^	2/87 (2.4%)	*p*-value (Fisher exact test) = 0.062; OR = 4.78

Comparisons between groups were conducted using Chi-square or Fisher’s exact tests for categorical variables and Student’s *t*-test or Mann–Whitney *U* test for continuous variables depending on data distribution. Statistically significant or higher trend associations between categorical variables were quantified using odds ratios (ORs) with corresponding 95% confidence intervals (CIs). Abbreviations: ASM, antiseizure medications; GTCS, generalized tonic–clonic seizures; AATS, absence or atypical absence seizures; TS, tonic seizures; MS, myoclonic seizures; SE, status epilepticus; GFAP, global functional assessement of the patient; MRI, magnetic resonance image; ICU, intensive care unit; PS, Partial or Focal Seizures; ES, epileptic spasms. A *p*-value < 0.05 was considered statistically significant (*); ^t^, means trend to.

**Table 7:** Comparison of epilepsy characteristics in WHS across key published studies.

Characteristics	Ho et al., 2018 [14]	Paprocka et al., 2022 [12]	Horiguchi et al., 2023 [13]	This Study, 2025
Study design	Retrospective; self-reported forms and clinical records	Systematic review of PubMed-indexed articles	Retrospective chart review	Retrospective; self-reported forms and clinical record review
Country	USA	Various English-language publications	Japan	Spain and Latin America
Sample size	141	56 studies included	11	140
Mean age at epilepsy onset (months)	10.9	6–12	9	9.8
Epilepsy frequency (%)	Only included epilepsy cases	~90%	100% (all patients had epilepsy)	91.3%
SE frequency (%)	50%	50%	10/11 (91%)	58.4%
Seizure types (in decreasing frequency)	AATS, GTCS, SE, TS, CS, MS, FS	GTCS, AATS, TS, SE, MS	GTCS, FS, MS, AATS	GTCS, AATS, FS, TS, MS, SE
Most commonly used ASMs (in decreasing frequency)	LEV, PB, VPA, DZP, CBZ, LMT DPH, CBZ, OXC poorly effective	Referenced by Ho et al., 2018 [14] Also mention ZNS, TPM (poor tolerability)	VPA, PB, LEV, BRV, LCM LEV, effective; PB, poorly effective	VPA, LEV, CLB/CLZ, LMT, OXC/CBZ, TPM, ETS, DZP

Abbreviations: GTCS, generalized tonic–clonic seizures; AATS, absence or atypical absence seizures; FS, focal seizures; TS, tonic seizures; MS, myoclonic seizures; SE, status epilepticus; CS, clonic seizures; VPA, valproic acid; LEV, levetiracetam; PB, phenobarbital; BRV, brivaracetam; LCM, lacosamide; CBZ, carbamazepine; OXC, oxcarbazepine; LMT, lamotrigine; DZP, diazepam; TPM, topiramate; ZNS, zonisamide; ETS, ethosuximide; CLB/CLZ, clobazam/clonaze; DPH, diphenylhidantoine or phenytoine; ~, around.

## Data Availability

We deposited data from SNP-arrays in the DECIPHER database (v11.33) repository (#562946 to 563086; IMMGWHS1-140).

## Supplementary Materials

The following are available online at https://www.mdpi.com/article/10.3390/jcm14228044/s1, Complementary results. Supplementary Table S1 is a disaggregated comparison between data sub-popupations used in the study.

## References

[1] Battaglia A., Carey J.C., Wright T.J. (2001). Wolf-Hirschhorn (4p-) syndrome. Adv. Pediatr..

[2] Battaglia A., Carey J.C., South S.T. (2015). Wolf-Hirschhorn syndrome: A review and update. Am. J. Med. Genet. C Semin. Med. Genet..

[3] Battaglia A., Carey J.C. (2021). The delineation of the Wolf-Hirschhorn syndrome over six decades: Illustration of the ongoing advances in phenotype analysis and cytogenomic technology. Am. J. Med. Genet. Part A.

[4] Blanco-Lago R., Málaga-Diéguez I., Granizo-Martínez J.J., Carrera-García L., Barrúz-Galián P., Lapunzina P., Nevado-Blanco J. (2017). En Representación Del Grupo Colaborativo Para El Estudio Del Sindrome de Wolf-Hirschhorn. Descripción de una cohorte española de 51 casos y revisión de la bibliografía [Wolf-Hirschhorn syndrome. Description of a Spanish cohort of 51 cases and a literature review]. Rev. Neurol..

[5] Zollino M., Murdolo M., Marangi G., Pecile V., Galasso C., Mazzanti L., Neri G. (2008). On the nosology and pathogenesis of Wolf-Hirschhorn syndrome: Genotype-phenotype correlation analysis of 80 patients and literature review. Am. J. Med. Genet. C.

[6] Nevado J., Ho K.S., Zollino M., Blanco R., Cobaleda C., Golzio C., Beaudry-Bellefeuille I., Berrocoso S., Limeres J., Barrúz P. (2020). International meeting on Wolf-Hirschhorn syndrome: Update on the nosology and new insights on the pathogenic mechanisms for seizures and growth delay. Am. J. Med. Genet. Part A.

[7] Kagitani-Shimono K., Imai K., Otani K., Kamio N., Okinaga T., Toribe Y., Suzuki Y., Ozono K. (2005). Epilepsy in Wolf-Hirschhorn syndrome (4p-). Epilepsia.

[8] Battaglia A., Filippi T., South S.T., Carey J.C. (2009). Spectrum of epilepsy and electroencephalogram patterns in Wolf-Hirschhorn syndrome: Experience with 87 patients. Dev. Med. Child Neurol..

[9] Ho K.S., South S.T., Lortz A., Hensel C.H., Sdano M.R., Vanzo R.J., Martin M.M., Peiffer A., Lambert C.G., Calhoun A. (2016). Chromosomal microarray testing identifies a 4p terminal region associated with seizures in Wolf-Hirschhorn syndrome. J. Med. Genet..

[10] Nevado J., Blanco-Lago R., Bel-Fenellós C., Hernández A., Mori-Álvarez M.A., Biencinto-López C., Málaga I., Pachajoa H., Mansilla E., García-Santiago F.A. (2025). Clinician-Based Functional Scoring and Genomic Insights for Prognostic Stratification in Wolf–Hirschhorn Syndrome. Genes.

[11] Blanco-Lago R., da Silva-Mori X., Bel-Fenellós C., Málaga-Diéguez I., Mori-Alvarez M.L.Á., Graña-Barreiro N., Lapunzina-Badía P., Nevado-Blanco J. (2022). Prevalence and geographic distribution of the Wolf-Hirschhorn syndrome in Spain. Rev. Esp. Salud Publica.

[12] Paprocka J., Kaminiów K., Yetkin O., Tekturk P., Baykan B., Leiz S., Kluger G., Striano P. (2024). Clinical and epilepsy characteristics in Wolf-Hirschhorn syndrome (4p-): A review. Seizure.

[13] Horiguchi A., Koichihara R., Kikuchi K., Nonoyama H., Daida A., Oba D., Hirata Y., Matsuura R., Ohashi H., Hamano S.I. (2023). Efficacy of Antiseizure Medications in Wolf-Hirschhorn Syndrome. Neuropediatrics.

[14] Ho K.S., Markham L.M., Twede H., Lortz A., Olson L.M., Sheng X., Weng C., Wassman E.R., Newcomb T., Wassman E.R. (2018). A survey of antiepileptic drug responses identifies drugs with potential efficacy for seizure control in Wolf-Hirschhorn syndrome. Epilepsy Behav..

[15] Corrêa T., Mergener R., Leite J.C.L., Galera M.F., Moreira L.M.D.A., Vargas J.E., Riegel M. (2018). Cytogenomic Integrative Network Analysis of the Critical Region Associated with Wolf-Hirschhorn Syndrome. BioMed Res. Int..

[16] Nakano Y., Fujita M., Ogino K., Saint-Amant L., Kinoshita T., Oda Y., Hirata H. (2010). Biogenesis of GPI-anchored proteins is essential for surface expression of sodium channels in zebrafish Rohon-Beard neurons to respond to mechanosensory stimulation. Development.

[17] Hastie T., Tibshirani R., Friedman J. (2001). The Elements of Statistical Learning: Hierarchical Clustering. Stat. Sci..

[18] Nishi A., Kuroiwa M., Shuto T. (2011). Mechanisms for the modulation of dopamine D1 receptor signalling in striatal neurons. Front. Neuroanat..

[19] Missale C., Nash S.R., Robinson S.W., Jaber M., Caron M.G. (1998). Dopamine receptors: From structure to function. Physiol. Rev..

[20] Bozzi Y., Dunleavy M., Henshall D.C. (2013). The role of dopamine signaling in epileptogenesis. Front. Cell Neurosci..

[21] Vallespín E., Palomares-Bralo M., Mori M.Á., Martín R., García-Miñaúr S., Fernández L., de Torres M.L., García-Santiago F., Mansilla E., Santos F. (2013). Customized high resolution CGH-array for clinical diagnosis reveals additional genomic imbalances in previous well-defined pathological samples. Am. J. Med. Genet. Part A.

