# Supplementary material for: Epilepsy in Wolf–Hirschhorn Syndrome: Clinical Insights from a Pediatric Cohort and a Review of the Literature
Source: J Clin Med. 2025 Nov 13;14(22):8044. doi: 10.3390/jcm14228044 (PMC12653090; doi:10.3390/jcm14228044)
Supplement: Supplementary file 1 [file jcm-14-08044-s001.zip › Supplemental Data text.pdf]

2. Complementary RESULTS.

2.2. Subpopulation Analysis. Statistically significant disaggregated-data

Despite some significant differences in some items, most clinical variables (see Supplemental data in Excel format with descriptive and frequency data) were similar between the two subpopulations (Pearson and Spearman analysis). Negative correlations indicate higher frequency in the Spanish group, while positive correlations reflect greater frequency in the Latin American group. Briefly, we highlight "*Seizure types*" more frequent in Spaniards, included "*myoclonic* and *focal seizures*" (r = –0.234, p =0.006; r = –0.175, p =0.043; Pearson analysis, respectively, and $\chi^2 = 4.107$, $df = 1$, $p = 0.043$, $\chi^2 = 7.350$, $df = 1$, $p = 0.007$). In the other way, the use of anti-seizure medications ("*to take ASMs*") was higher in Latin America (r = 0.262, $p = 0.002$, and $\chi^2 = 9.274$, $df = 1$, $p = 0.003$), particularly clobazam/clonazepam (r = 0.362, $p < 0.0001$, and $\chi^2 = 17.721$, $df = 1$, $p < 0.0001$) and "*other ASMs*" (such as phenytoin, or phenobarbital; r = 0.441, $p < 0.0001$ and $\chi^2 = 26.279$, $df = 1$, $p < 0.0001$).

Additional associations, such as brain malformations on MRI were more frequent in the Latin American group (r = 0.177, p = 0.048). The mortality was slightly higher in the Spaniards (negative correlation: r = –0.229, p = 0.006). Deaths were not reported in the Latin American cohort. However, this likely reflects incomplete data collection rather than a true absence of cases, as we are aware of deceased individuals in this group. The suspicion of diagnosis prior to genetic confirmation was more common in Latin America (r = 0.187, p = 0.027). Cognitive variables also differed slightly, with higher rates of diaper use (r = 0.220, p = 0.010) and lower frequency of sentence production (r = –0.254, p = 0.003) in the Latin American cohort.

2.8. Status epilepticus with ICU management (ER-status)

Bivariate correlation analyses using Pearson and Spearman coefficients was also conducted to examine associations among status visiting ICU, called, "*ER-status*" and several fetures. Thus, status visiting ICU correlated positively with: i) seizure presence (r = 0.232, p = 0.007), non-febrile seizures (r = 0.250, p = 0.003), seizure subtypes: GTCS (r = 0.259, p = 0.027), myoclonic seizures (MS, r = 0.181, p = 0.037), and epileptic spasms (ES, r = 0.210, p = 0.015), deletion size (r = 0.280, p = 0.012), GFAP (r = 0.483, $p < 0.0001$) and its intermediates (r: 0.239, p = 0.005; r = 0.308, p=<0.0001; r = 0.269, p=0.002), regarding developmental delay and comorbidities, respectively. As well as the use of ASMs (r = 0.308, $p < 0.0001$), notably; VPA (r = 0.368, $p < 0.0001$), CLB/CLZ (r = 0.202), ethosuximide (ETS, r = 0.174, p = 0.044), and other ASMs (r = 0.257, p = 0.003). With number of status episodes (r = 0.727, $p < 0.0001$), number of ASMs (r = 0.411, $p < 0.0001$), and maximum number of simultaneous ASMs (r = 0.363, $p < 0.0001$). Comorbidities included associations with gastrostomy (r = 0.217, p = 0.012), auditory anomalies (r = 0.211, p = 0.014), and MRI-detected malformations (r = 0.200, p = 0.022). Negative associations were found with: ii) walking with help (r = –0.214, p=0.013) and unaided (r = –0.201, p=0.02), seizure control (r = –0.206, p = 0.016), motor development (r = –0.190, p = 0.028), cognitive performance (r = –0.175, p = 0.042), and height at birth (r = –0.183, p = 0.033)

We also observed that the differences between clusters regarding "*ER-status*" were accompanied by a broader clinical profile of increased severity. Specifically, higher cluster severity was associated with a greater number of status episodes, a higher total number of anti-seizure medications (ASMs) used, and an increased maximum number of ASMs employed concurrently. These clusters also included more patients experiencing non-febrile seizures and showed significantly worse "functional" (measured by GFAP variable) outcomes. These variables were inversely correlated with favorable indicators such as effective seizure control, and the proportion of patients who had discontinued ASM treatments. In line with this, we found a statistically significant inverse association between cluster severity and cognitive or motor function, suggesting more profound impairment in these developmental domains.

2.4.4. Age and Status Epilepticus visiting ICU

A similar pattern to "*status*" emerged when evaluating ICU-status epilepticus (*ERStatus*). Cluster 2 again showed the highest percentage of cases (50.0%), followed by Cluster 3 (40.8%) and Cluster 1 (25.0%). Notably, the youngest patients did not show a disproportionately higher rate of *ERStatus*, and the oldest cluster had the lowest. Analysis of the mean number of status episodes per cluster did not reveal statistically significant differences either. However, there was a trend toward a greater number of episodes in older patients, with Cluster 1 averaging 7.25 episodes compared to 3.00 in Cluster 2 and 2.73 in Cluster 3.

2.4. **Table S1 Supplemental data**. Overall functional status clusters by using the variable "*GFAP score*".

"GFAP"	**Cluster 4**	**Cluster 3**	**Cluster 2**	**Cluster 1**
GFAP (value)	108.24±20.57(113) *<br>range 64-136	185.39±21.57(188<br>)**<br>range 149-221	261.21±20.65(260) ***<br>range 225-301	342.14±27.44(336)<br>range 303-410
size of deletion (Mb)	3.32±2.49 (2.9) *<br>range 0.01-9.50	7.47±5.36 (5.55) **<br>range 1.30-23.73	10.01±6.12 (8.92) ***<br>range 1.39-27.30	12.52±5.27(12)<br>range 5.94-23.90
Global Epilepsy items	25.00±14.96(23) *<br>range 0-60	54.05±17.84(57) **<br>range 0-86	82.75±19.46(82) ***<br>range 42-135	103.90±27.44(100)<br>range 45-170
Age of seizures (months)	11.57±11.38 (11.5)<br>range 0.01-36	9.93±6.02 (9)<br>range 0-30	9.99±5.30 (9)<br>range 2-30	7.09±4.15 (8)<br>range 0.01-14
Seizures w fever	11/20(55.00%) *	29/40 (72.50%)	42/53 (79.24%)	13/21 (61.90%)
Seizures w/o fever	3/20(15.00%) *	22/40 (55.00%) **	37/53 (69.80%) ***	19/21(90.47%)
Status	3/20(15.00%) *	15/40 (37.50%) **	46/53(86.79%)	16/21 (76.19%)
Number status	0.85±2.64 (0) **<br>range 0-11	1.18±3.30 (0) **<br>range 0-20	5.13±9.15 (2)<br>range 0-55	3.48±3.08 (3)<br>range 0-10
Status to ICU	2/20(10.00%) **	8/40 (20.00%) **	35/53(66.03%)	13/21 (61.90%)
Take<br>ASM, now	11/20(55.00%) *	33/40 (82.50%) **	51/53 (96.22%)	20/21(95.23%)
Number of ASM	1.22±1.34 (1) **<br>range 0-5	1.63±1.21 (1) **<br>range 0-4	2.62±1.37 (2)<br>range 0-6	2.81±1.29 (3)<br>range 0-5
Monotherapy	6/20(30.00%)	26/40 (65.00%) **;@	24/53 (45.28%)	7/21 (33.33%)
Maximum number of ASM used simultaneously	0.85±0.81 (1)<br>range 0-2	1.33±0.76 (1)<br>range 0-3	1.81±0.71 (2)<br>range 0-3	2.29±1.15 (2)<br>range 0-5
Took ASM, not now	7/20 (35.0%) *	8/40 (20.0%) **	4/53 (7.54%)	1/21 (4.76%)
Crisis control (1 up to 6)	5.24±0.77 (5) **<br>range 3-6	3.95±1.52 (5) **<br>range 1-6	3.40±1.45 (3) ***<br>range 1-5	1.95±1.07 (2)<br>range 1-4
Seizures	13/21 (61.90%) *	39/41 (95.12%)	53/53 (100%)	21/21 (100%)

ASM, anti-seizure medication; GFAP, global functional assessement of the patient; ICU, intentive care unit; &) see Table 4 for explanation*) $p < 0.05$ vs CL3, CL2, CL1; **) $p < 0.05$ vs CL2, CL1; ***) $p < 0.05$ vs CL1; $) $p < 0.05$ vs CL3, CL2.

The data reveals significant differences in epilepsy-related variables, with notable patterns based on GFAP values and deletion sizes across clusters. Unsupervised clustering based on the “GFAP” variable revealed four distinct clinical subgroups with a clear gradient of severity. Cluster 1 encompassed the most severe cases, marked by high GFAP scores, large 4p deletions, early seizure onset, frequent status epilepticus episodes, poor seizure control, and a higher incidence of intensive care admissions. Cluster 2 also reflected a severe phenotype but with slightly better seizure control and fewer status events compared to Cluster 1. Cluster 3 represented an intermediate severity profile, showing moderate GFAP scores, smaller deletions, and improved seizure control relative to the first two clusters. Finally, Cluster 4 corresponded to the mildest clinical presentation, characterized by later onset of seizures, fewer status episodes, and the most favorable seizure control across the entire cohort.
